# Surveillance of central-line associated bloodstream infections in ICU at a Malaysian medical centre

**DOI:** 10.1186/1753-6561-5-S6-P212

**Published:** 2011-06-29

**Authors:** A Sulong, N Awang Jalil, R Ramli, M Mohd Yusoff

**Affiliations:** 1 Dept of Medical Microbiology & Immunology, Faculty of Medicine, Universiti Kebangsaan Malaysia Medical Centre, Kuala Lumpur, Malaysia; 2 Dept of Anaesthesiology, Faculty of Medicine, Universiti Kebangsaan Malaysia Medical Centre, Kuala Lumpur, Malaysia

## Introduction / objectives

We conducted a prospective surveillance study to determine the incidence and epidemiology of CLABSI in the intensive care unit (ICU) at a tertiary teaching medical centre using CDC definition of a bloodstream infection that develops in a patient who had a central line at the time of, or within 48 hours before, the onset of the infection.

## Methods

A cross section observational study was conducted in a 24-bed GICU over 10-months period from April 2008 until January 2009 among patients with central venous catheters (CVCs) inserted in the ICU or operation theatre.

## Results

A total of 20 CLABSI cases were identified among 155 CVCs in 100 patients with 3106 catheter days. The overall rate of CLABSI was 6.4 per 1000 catheter-days and device-utilization (DU) ratio of 0.81. The mean length of ICU stay for CLABSI and non- CLABSI cases was 37.2 days and 17.4 days respectively, while the median length of ICU stay for CLABSI cases was 16.0 days and for non- CLABSI cases was 10.0 days. This contributed to 6 extra days of ICU stay in CLABSI cases. CVCs inserted via the femoral vein were associated with higher infection rate of 22.2% followed by those of internal jugular vein (15.4%) and subclavian vein (5.1%). Gram-negative bacteria accounted for 50% of the CLABSI cases whereas gram-positive cocci and fungi caused 35% and 15% of these infections respectively.

## Conclusion

Both of the CLABSI rate of 6.4 per 1000 catheter-days and DU ratio of 0.81 were above the 90^th^ percentile of the NHSN benchmark. Comparing our findings to the INNIC 2003-2008 studies with 7.4 CLABSI per 1000 catheter-days, our CLABSI rate was lower. However, our DU ratio was higher. Hence, interventions aimed at improving outcomes related to CVCs should seriously be considered.

## Disclosure of interest

None declared.

